# Hepatobiliary Events in Migraine Therapy with Herbs—The Case of Petadolex, A Petasites Hybridus Extract

**DOI:** 10.3390/jcm8050652

**Published:** 2019-05-10

**Authors:** Nora Anderson, Jürgen Borlak

**Affiliations:** Hannover Medical School, Centre for Pharmacology and Toxicology, Carl-Neuberg-Str. 1, 30625 Hannover, Germany; nora.anderson@web.de

**Keywords:** herbal-induced liver injury, butterbur extract, pharmacovigilance, clinical and animal liver pathology

## Abstract

Petadolex^®^, a defined butterbur extract has clinically proven efficacy against migraine attacks. However, spontaneous reports indicate cases of herbal induced liver injury (HILI). While most HILI patients presented mild serum biochemistry changes (<3 ULN, dose range 50 to 225 mg/day; treatment duration 4–730 days) nine developed severe HILI (average time-to-onset 103 days, ALT-range 3–153; AST 2–104-fold ULN). HILI cases resolved after medication withdrawal though two patients required liver transplantation. Liver biopsies revealed an inconsistent injury pattern, i.e. necrosis, macrovesicular steatosis, inflammation, cholestasis, and bile duct proliferation. Causality assessment rated 3 cases likely, 13 possible, 8 unlikely and 24 as unclassifiable/unclassified. Note, 22 patients reported hepatotoxic co-medications especially during periods of pain. A no-observable-adverse-effect-level at 15-fold of the maximal clinical dose (3 mg/kg/day MCD) was established for rats. At >45 and 90-fold MCD bile duct hyperplasia was observed but could not be confirmed in an explorative minipig study at 218-fold MCD. Human hepatocyte studies at 49-fold C_max_ serum petasins (=active ingredient) and therapeutic Ibuprofen, Paracetamol and Naratriptan concentrations evidenced liver transaminase and CYP-monooxygenase changes. Collectively, Petadolex^®^ HILI cases are rare, idiosyncratic and frequently confounded by co-medications. A physician-supervised self-medication plan with herbs and pain relief medication is needed to minimize risk for HILI.

## 1. Introduction

Herbal and Dietary Supplements (HDS) are a multi-billion dollar enterprise and are considered to be safe by the public. However, the U.S. Drug-Induced-Liver Injury-Network reported HDS related liver injury to be on the rise accounting for about 20% of all DILI cases [1]. There is unmet need to better diagnose herb induced liver injury (HILI) and to provide guidance for its clinical management. 

We report the case of Petadolex^®^, a defined *Petasites hybridus* (butterbur) extract used as migraine preventive treatment in adults. The extract is primarily composed of sesquiterpene esters, mainly of the petasin and the furanopetasin chemotype [2] and constitutes a mixture of petasin, isopetasin, neopetasin as well as furanoeremophilanes and eremophilanlactones. In the following this mixture is referred to as ‘petasin’. Petadolex^®^ is specified to ≥15% petasin, i.e. the active ingredient of butterbur and was introduced in Germany in 1972 and in the US in 1998. Its clinical efficacy in migraine therapy was evidenced in placebo-controlled double-blinded clinical investigations. A significant reduction in the frequency of migraine attacks was reported [3,4] particularly after four months of treatment [5,6]. In 2012, the American Academy of Neurology concluded butterbur to be effective for migraine prevention [7]. However, post marketing surveillance in Europe revealed HILI cases, which prompted concern over its safe use. Note, butterbur contains pyrrolizidine alkaloids (PAs) and once metabolically activated [8] can damage sinusoidal endothelium resulting in sinusoidal obstruction syndrome (SOS) [9,10]. 

Our study aimed to (1) evaluate the causality of spontaneously reported hepatobiliary ADRs involving Petadolex^®^ (2) to compare liver biopsy findings of suspected HILI cases with injury patterns observed in rats and minipigs after repeated oral dosing and (3) to assess hepatotoxicity in primary human hepatocyte (PHH) cultures treated with Petadolex^®^ and combinations of NSAIDs, Paracetamol and/or triptanes commonly used in the treatment of migraine attacks.

## 2. Materials and Methods

### 2.1. Petasites Hybridus Extracts

Certified product 056180 (Extract A, 37% petasin), 096120 (Extract B, <1% petasin) and 056180/096120 (Extract C = Petadolex^®^, 19.1% petasin) were supplied by Dr. Koch, Weber & Weber International GmbH & Co. KG, Inning/Ammersee, Germany. PHH studies were performed with Paracetamol (CAS: 103-90-2, Sigma-Aldrich, Germany), Ibuprofen (Alpha-Methyl-4-(isobutyl)phenylacetic acid, CAS: 31121-93-4, Sigma-Aldrich, Germany) and Naratriptan (N-methyl-2-[3-(1-methylpiperidin-4-yl)-1H-indol-5-yl] ethanesulfonamide hydrochlorid, CAS: 143388-64-1, GlaxoSmithKline, Germany). DMSO was used as vehicle (final concentration in cell culture <0.5%). 

### 2.2. Pharmacovigilance—Periodic Safety Updated Reports (PSUR)

Periodic safety update reports (PSUR) were provided by Weber & Weber International with the aim to assess causality independently. A total of 60 hepatobiliary ADRs were reported and included 42 female, 16 male and two unspecified cases. Information regarding the 48 spontaneously reported HILI cases is given in Table 1. Furthermore, Table 2 compiles 12 cases recorded during clinical trials.

Patients provided informed consent prior to the study entry and details of the clinical trials are given in [3,4,5,6]. The HILI cases were evaluated according to the WHO-UMC system by an expert panel consisting of specialists in internal medicine/neurology, board certified pathologist and clinical pharmacologist and toxicologist. There was agreement among the reviewers in the grading of cases. Independently, a subset of Petadolex^®^ suspecting severe HILI cases was evaluated by hepatologists/gastroenterologists using the RUCAM method. The findings were reported in a journal which is not indexed in Medline [11]. 

### 2.3. Toxicity Studies in Animals

The results of an acute and repeated oral dose good laboratory practice (GLP) compliant toxicology study in rats were previously published [12]. The studies were carried out at Harlan Bioservices for Science GmbH, Walsrode, Germany, in accordance with the EEC Council Directives 65/65 and 73/318 and subsequent amendments detailed in the Rules Governing Medicinal Products in the European Union, the ICH Guideline S4 for Toxicity Testing (Proceedings of the First International Conference on Harmonization 1991), EEC Directive 92/69/EEC (31 July 1992), Annex V, Test B1 (20 December 1992), and the Organisation for Economic Co-operation and Development Guideline 401. 

In the six-month chronic toxicity study the doses were >15-, 45 and 90-fold of the maximum clinical dose (MCD), i.e. 3 mg/kg/day. Note, the histopathology and serum biochemistries of animals four weeks after treatment termination (“recovery animals”) were not reported so far. 

Additionally, an explorative study with *n* = 6 Göttingen minipigs weighing approximate 25 kg was performed at the LPT research facility in Hamburg, Germany. The study was not GLP compliant but followed the principles of Good Laboratory Practice Regulations of the EC as well as the OECD Principles of Good Laboratory Practice’ Document Nos. 1, 8 and 13 ENV/MC/CHEM (98) 17, ENV/JM/MONO (99) 24 and ENV/JM/MONO (2002) 9, respectively.

All experimental protocols were approved by the Lower Saxony State Office for Consumer Protection and Food Safety and the Office for Health and Consumer protection of the city of Hamburg, Germany.

Two different butterbur extracts defined by their low (0.84%) and rich petasin content (16.1%) were evaluated. Dose levels of 60, 180, 360 and 720 mg/kg body weight were administered by oral gavage for three consecutive days. No signs of toxicity were observed. Subsequently, a 28-day repeated oral dose study was completed at 218-fold MCD. 

### 2.4. Studies with Primary Human Hepatocyte Cultures

The use of human liver resection material was approved by the Ethical Committee of Hannover Medical School; patients had given informed consent. Supplementary Table S1 provides basic patient characteristics. Isolation and culture of human hepatocyte cultures was done as previously reported [12]. All experiments were performed in accordance with relevant guidelines and regulations.

### 2.5. Treatment of Hepatocyte Cultures

Hepatocyte cultures were treated with extracts B and C at 60 ng/mL and 15 µg/mL and extract A at 10 µg/mL for 72 h. Due to differences in petasin content, the concentration range tested in hepatocyte cultures was 11.5–3700 ng/mL petasin. Specifically, extract C contains 19.1% petasin and assays performed at 15 µg/mL are equal to 2865 ng/mL petasin, i.e. 15,000 ng/mL × 19.1% = 2865 ng/mL petasin. Given that petasin is the major active ingredient of butterbur, the petasin concentrations can also be related to human PK data. For instance, an oral dose of either 116 mg or 232 mg butterbur extract Ze339 given to 24 healthy volunteers yielded C_max_ petasin serum concentrations of 25.5 ± 14.8 and 58.1 ± 26.7 ng/mL, respectively [13]. Correspondingly, the 15 µg/mL extract C tested in hepatocyte cultures is equal to >114 or >49-fold C_max_ petasin concentrations based on a dose of 116 mg or 232 mg butterbur extract. At the 15 µg/mL extract C concentration, a 30% reduction in cell viability was determined in the MTT assay after daily dosing for 72 h. To assess possible interactions, hepatocyte cultures were treated with combinations of Petadolex^®^ and therapeutic C_max_ Paracetamol (20 μg/mL), Ibuprofen (30 μg/mL) or Naratriptan (10 ng/mL) concentrations.

### 2.6. Assays to Determine Cell Viability and Metabolic Competence

Cell viability and mitochondrial toxicity was determined by the MTT assay [12]; urea, ALT, AST and GGT were measured on a Cobas FARA clinical chemistry analyser (Roche, Germany). CYP450 monooxygenase activity was assayed with testosterone as a substrate; the metabolites were quantified by HPLC-UV [12]. 

### 2.7. Statistical Analyses

Statistical significance testing was performed with the GraphPad Prism software version 6.05 (GraphPad Software, San Diego, CA, USA). The Shapiro–Wilk test for normality was applied to experimental data. Depending on the distribution of the data, the Student’s *t*-test or the non-parametric Wilcoxon Rank test was employed. The Fisher exact test was used to determine significant differences in dosage related bile duct proliferations and its reversibility in animals after treatment cessation.

## 3. Results

### 3.1. Pharmacovigilance of HILI Cases

Until 2015, a total of 60 hepatobiliary ADRs were reported and included 42 female, 16 male and two unspecified cases. Given the higher liability for migraine in women, the observed gender bias is in line with expectations [14]. Information regarding the 48 spontaneously reported HILI cases is given in Table 1. Furthermore, Table 2 compiles 12 cases recorded during clinical trials with 397 patients on Petadolex^®^. Importantly, none of the cases reported from clinical trials were serious and involved very minor changes in serum biochemistries (range ALT 0.6 to 1.2; AST 0.3 to 1.3 of ULN, Table 2). 

The majority of spontaneously reported HILI cases (Table 1) presented ≤3-fold ULN serum biochemistry changes (ALT, AST and occasionally GGT, urea and antibody titres). Causality assessment revealed 3 cases as likely, 13 as possible, 8 as unlikely and 24 as unclassifiable/unclassified. However, nine females and one male patient presented marked changes in serum biochemistries (ALT 3-150-fold ULN; AST 2-104-fold) with two patients requiring liver transplantation. More detailed information of severe HILI cases is given in Table 3 and for three cases a relationship between exposure to Petadolex^®^ and hepatobiliary ADR was questioned by the expert panel.

Specifically, case #23 occurred 14 days after surgery and blood transfusion; total Petadolex^®^ intake was 3 to 6 days. Based on the long-lasting medication experience with Petadolex^®^ this would be an unprecedented case and may possibly arise from anaesthesia or blood transfusion. Moreover, patient #21 received tamoxifen for the treatment of breast cancer; an increased risk for NASH has been reported for this drug [15]. Patient #17 displayed marked serum transaminase elevations with extensive weight loss and a medical history of thoracic pain episodes prior to treatment with Petadolex^®^. Additionally, patients #12, #39 and #30 were positive for antinuclear autoantibodies to conceivable indicate an autoimmune hepatitis that may have been unmasked by Petadolex^®^ treatment. Notwithstanding, histopathology of case #12 was judged atypical for autoimmune hepatitis and case #39 showed a chronic hepatic necro-inflammatory disorder, which is consistent with the morphological feature of an autoimmune hepatitis. This patient presented a medical history of severe hepatitis prior to Petadolex^®^ treatment 10 years ago. A second episode of hepatitis was associated with jaundice and arthralgia and occurred two months after tonsillectomy. Hypersensitivity resulting from anaesthesia was suspected as a cause for hepatitis. The patient went into encephalopathy grade III and received a liver transplant within 1 week of hospital admission. Two cases were judged unlikely; case #48 was diagnosed with choledocholithiasis. The patient presented an isolated >46-fold bilirubin elevation due to bile flow obstruction with no change in liver transaminases. Moreover, case #9 was considered unlikely as hepatitis developed six weeks after Petadolex^®^ treatment cessation. 

To search for possible dose and/or time-to-onset relationships, scatter blots of serum biochemistries of suspected HILI cases were prepared. Cases were divided into cohorts of ≤3 and >3-fold ULN serum biochemistry changes. Except for ALT and the parameter time-to-onset at ≤3 ULN, no clear relationship could be ascertained (Supplementary Figure S1). Therefore, the cases are considered to be primarily idiosyncratic nature.

### 3.2. Co-Medications as A Possible Cause for Hepatobiliary ADRs

Among the suspected Petadolex^®^ HILI cases, 22 patients reported co-medication with drugs known to cause liver injury, such as pain relief and migraine prevention therapeutics, including amitriptyline and metoprolol. In particular NSAIDs are associated with risk of hepatic injury [16]. Of the severe HILI cases, 50% had additional migraine medication (*n* = 3 NSAIDs, *n* = 2 zolmitriptan as well as an unspecified herbal product) that may have contributed or even elicited liver injury. Patients #9 and #12 received contraceptives known to potentially cause cholestasis [17]. For another nine cases, co-medication with triptans was reported, which have been limited in their use due to modulation of CYP1A2 or MAOA activity [18,19]. In addition, eight patients reported Paracetamol alone or combinations of NSAIDs for pain relief, particularly during acute migraine attacks. 

### 3.3. Histopathology Findings of Severe HILI Cases 

Liver biopsy findings for eight severe HILI cases were available. An inconsistent pattern of mixed type injury was observed. Major findings were necrosis, macro-vesicular steatosis, cholestasis and fibrosis. Cases #7, #12, #23, #30 and #35 presented inflammation and inflammatory infiltrates as major finding; notwithstanding case #23 and #39 displayed bile duct proliferation. 

### 3.4. Results from A Chronic Toxicity Study in Rats

Absolute and relative organ weights were dose-related increased at mid and high doses after six months of treatment. Histopathology revealed bile duct hyperplasia at 45 and bile duct dilatation at 90-fold of MCD with male rats being more frequently affected.

The bile duct proliferation was corroborated by mild increases in plasma bilirubin and GGT. There was no clear pattern in the temporary alterations of serum biochemistries during the six-month chronic toxicity study with very slight but statistically insignificant changes of AST and ALT (Supplementary Table S2). These were reduced to approximately 90% of control values at the end of the study, while bilirubin was insignificantly increased by 4% in females and 85% in male rats, but with considerable variability amongst individual animals. Supplementary Table S2 summarizes clinical biochemistry data and major histopathology findings. Unlike severe HILI cases (see above), no evidence for necrosis, cholestasis or mixed type toxic liver injury was observed. However, mild lipidosis was noted in vehicle-treated control and high dose-treated animals. The vehicle Miglyol 812 is a medium change triglyceride and lipids derived from it may elicit serum biochemistry abnormalities and histocytosis, as reported by other investigators [20]. 

### 3.5. Liver Pathology Findings four Weeks after Treatment Cessation of Rats 

**Figure 1:**
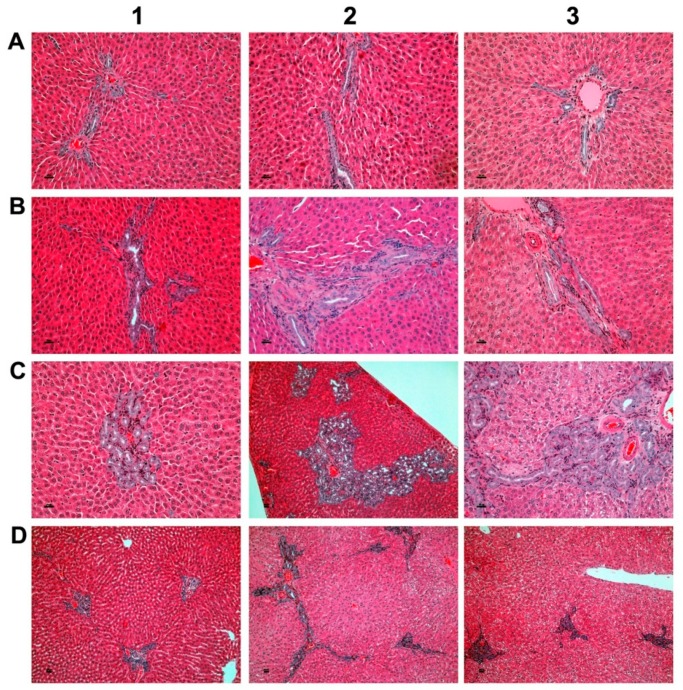
Bile duct hyperplasia after repeated oral dosing of rats with a *Petasites hybridus* extract (Petadolex) for six months. (**A**). HE staining of liver sections from control animals that were treated with the vehicle Migylol 812 for six months (A1, A2). Shown in A3 is a control recovery animal which also received vehicle treatment for six months, but was subsequently given a normal laboratory diet for four weeks. No lesions were observed. (**B**) Animals treated with the *Petasites hybridus* extract at 45-fold of maximum clinical doses. HE staining of liver sections revealed mild to moderate bile duct proliferations. (**C**) Animals treated with the *Petasites hybridus* extract at 90-fold of maximum clinical doses. HE staining of liver sections revealed moderate to marked bile duct proliferations. (**D**) Recovery animals were treated for six months with the *Petasites hybridus* extract at 90-fold of maximum clinical doses. Subsequently the recovery animals were given a normal laboratory diet for four weeks. Reversibility of bile duct proliferations was observed but did not resolve within the four weeks of the recovery period. All images are a magnification of 125× and were taken with a Nikon Ni-E microscope, Japan. Image capture was done with the Nikon NIS basic research microscopic imaging software version 4.3. The images were converted into tiff files and compiled in Adobe Photoshop version CS5.

As part of the chronic toxicity study, 40 rats per dose group were treated at >15-, 45 and 90-fold of the maximum clinical dose (MCD, i.e. 3 mg/kg/day) for six months. Figure 1 depicts examples of bile duct proliferations which were observed in the mid and high dose groups.

**Figure 2:**
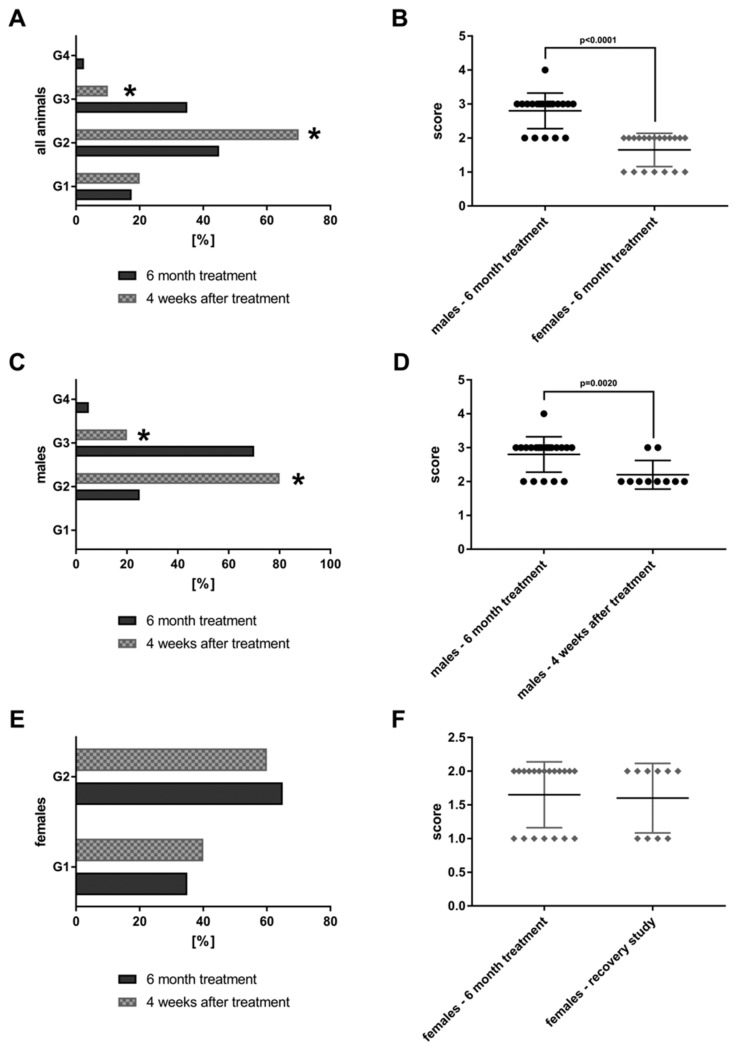
Bile duct proliferations after repeated oral dosing of rats with a *Petasites hybridus* extract (Petadolex) for six months and a recovery period of four weeks. Bile duct proliferations were dosage related with both sex. The grading of lesions ranges from minimal (G1), mild (G2), moderate (G3) to marked (G4). (**A**) Grading of bile duct proliferations of 40 high dose animals (dark column) and 20 recovery animals (grey patterned) treated for 6 month. Using the Fischer exact test, a statistically significant reduction from moderate (G3) to mild (G2) was calculated for animals which were allowed to recover from the treatment for four weeks (*p* = 0.0001). (**B**) The scores for male female animals after repeated treatment for six months are compared. Males are more sensitive to the treatment effects as determined by the Wilcoxon-Mann-Whitney-Test. (**C**) Grading of bile duct proliferations of 20 high dose males (dark column) and 10 male recovery animals (grey patterned) treated for six months. Using the Fischer exact test, a statistically significant reduction from moderate (G3) to mild (G2) was calculated for animals that were allowed to recover from the treatment for four weeks (*p* = 0.0161). (**D**) The scores for male animals after repeated treatment for six months and male recovery animals are compared. The grades of lesion differed significantly as determined by the Wilcoxon-Mann-Whitney-Test. (**E**) Grading of bile duct proliferations of 20 high dose females (dark column) and 10 female recovery animals (grey patterned) treated for six months. There was no difference between the groups, albeit the lesions are less pronounced when compared to males. (**F**) The scores for female animals after repeated treatment for six months and female recovery animals. There was no difference between the groups.

The findings are compared to 20 animals that were treated at 90-fold MCD for six months but were allowed to recover from the treatment for a period of four weeks (“recovery animals”). Subsequently, the lesions were graded by a board certified veterinary pathologist in compliance with international rules of good laboratory practice (GLP). The lesions were also evaluated independently by a board certified human pathologist. The grading of lesions ranges from minimal (G1), mild (G2), moderate (G3) to marked (G4). The grades of 40 high dose animals and 20 recovery animals are shown in Figure 2.

Both sex bile duct proliferations were dosage related and tended to be reversible, at least in part. A statistically significant reduction from moderate (G3) to mild (G2) was determined using the Fischer exact test (*p* = 0.0001). Furthermore, the average score for bile duct proliferations differed significantly by gender (Figure 2B) with males presenting higher grades. Figure 2C,D show the grading for male animals only. Once again, a significant reduction from G3 to G2 is observed (*p* = 0.0161) after treatment cessation; the average scores are given in Figure 2D. Conversely there was no difference in the grading of bile duct proliferations of females when recovery animals are considered. However, the grades in females are significantly lower as compared to males (Figure 2B). 

Importantly, the bile duct proliferations occurred in the absence of significant serum biochemistry changes. Next to proliferation of the existing bile duct epithelium, metaplastic changes of periportal hepatocytes were observed to assist in bile drainage at excessive treatment doses. 

### 3.6. Explorative Studies with Minipigs 

Minipigs were treated by daily oral gavage for 28 days with a butterbur extract, either low (<1%) or rich in petasin (>16%) at 218-fold of MCD. No morphological changes in the liver were observed with any of the extracts; however, a two-fold increase in indirect bilirubin and an approximately 70% reduction in bile acid serum concentrations were noted. Other serum biochemistries, i.e., aPTT, ALT, AST, GGT and 5′-nucleotidase were unchanged after repeated oral dosing for 28 days. 

### 3.7. In vitro Hepatotoxicity Assays

**Figure 3:**
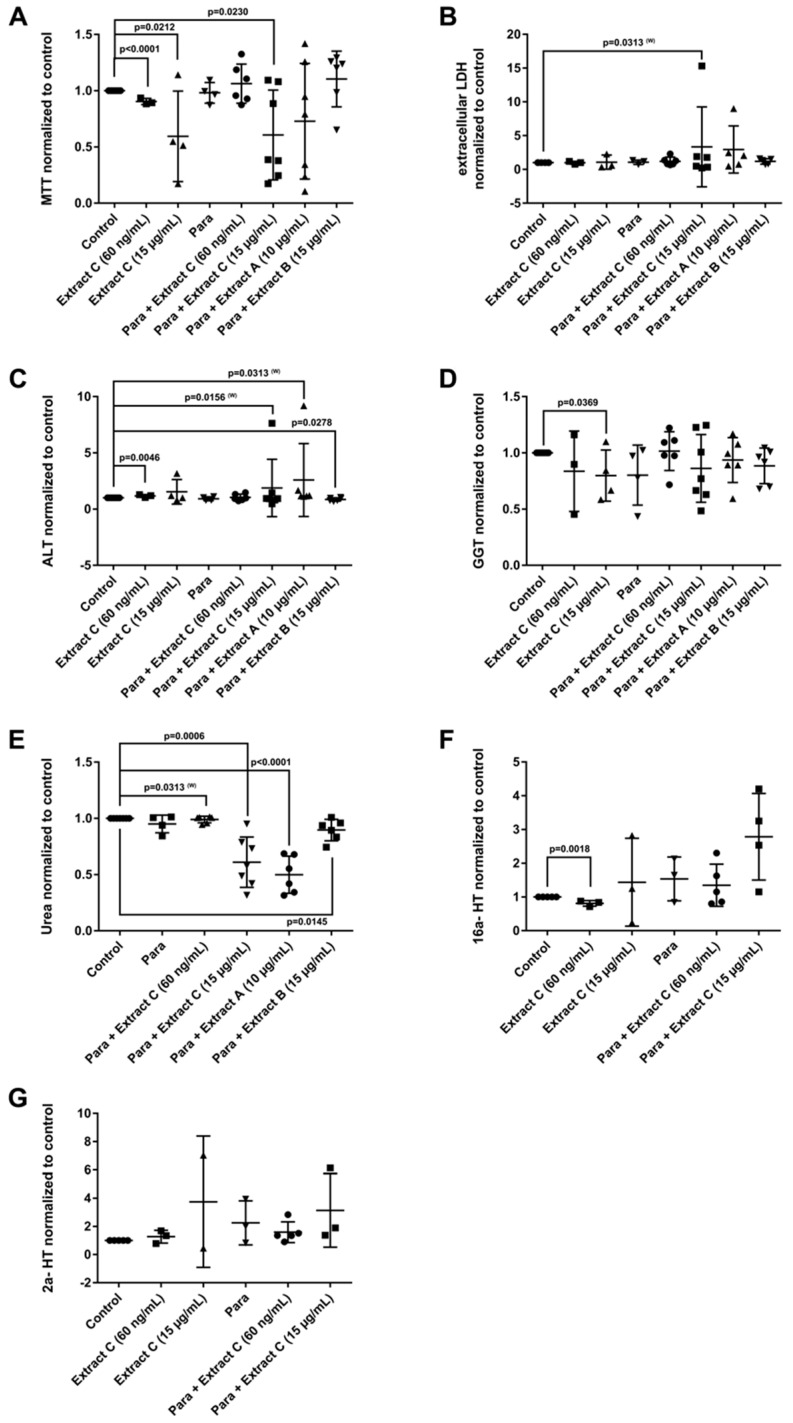
Effects of different *Petasites hybridus* extracts and Paracetamol on clinical liver function tests in cultures of human hepatocytes after daily treatment for 72 h. (**A**) MTT mitochondrial reductase activity. (**B**) LDH leakage/extracellular activity. (**C**) Alanine aminotransferase activity. (**D**) glutamyltransferase activity. (**E**) Urea production. (**F**,**G**) Testosterone 16 and 2 hydroxylation activity. The data of individual donors are given normalised to the DMSO vehicle control. The *Petasites hybridus* extracts A, B and C are specified to 37%, <1% and 19.1% petasin, respectively. Extract C is the marketed product Petadolex^®^ and human hepatocyte cultures were treated at therapeutic C_max_ (=60 ng/mL) and >49-fold therapeutic petasin C_max_ (=15 µg/mL) concentrations. Hepatocyte cultures were also treated with Paracetamol at therapeutic C_max_ (=Para; 20 µg/mL) concentrations and in combinations with different *Petasites hybridus* extracts. The data were analysed with the GraphPad Prism software version 6.05.

**Figure 4:**
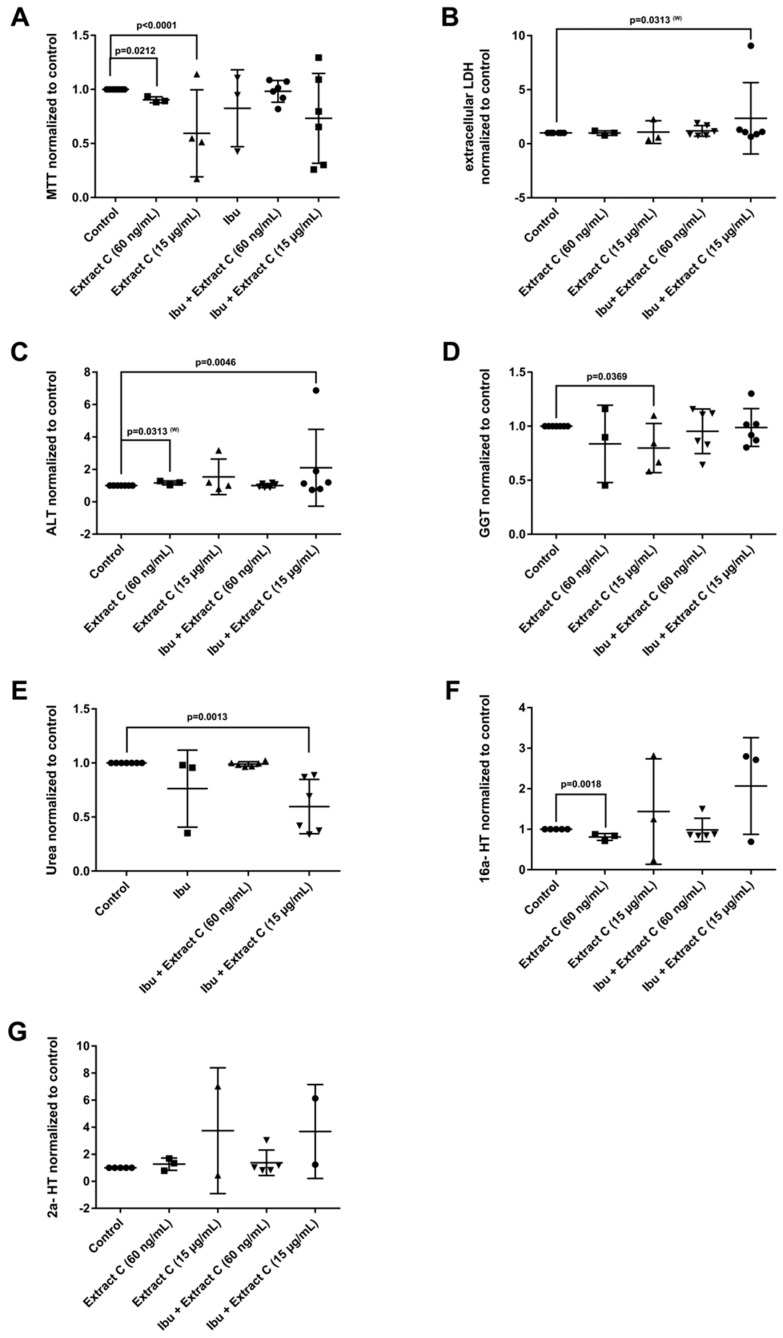
Effects of different *Petasites hybridus* extracts and Ibuprofen on clinical liver function tests in cultures of human hepatocytes after daily treatment for 72 h. (**A**) MTT mitochondrial reductase activity. (**B**) LDH activity. (**C**) Alanine aminotransferase activity. (**D**) Glutamyltransferase activity. (**E**) Urea production. (**F**,**G**) Testosterone 16 and 2 hydroxylation activity. The data of individual donors are given normalised to the DMSO vehicle control. The *Petasites hybridus* extracts A, B and C are specified to 37%, <1% and 19.1% petasin, respectively. Extract C is the marketed product Petadolex^®^ and human hepatocyte cultures were treated at therapeutic C_max_ (= 60 ng/mL) and >49-fold therapeutic petasin C_max_ (=15 µg/mL) concentrations. Hepatocyte cultures were also treated with Paracetamol at therapeutic C_max_ (=Para; 20 µg/mL) concentrations and in combinations with different *Petasites hybridus* extracts. The data were analysed with the GraphPad Prism software version 6.05.

**Figure 5:**
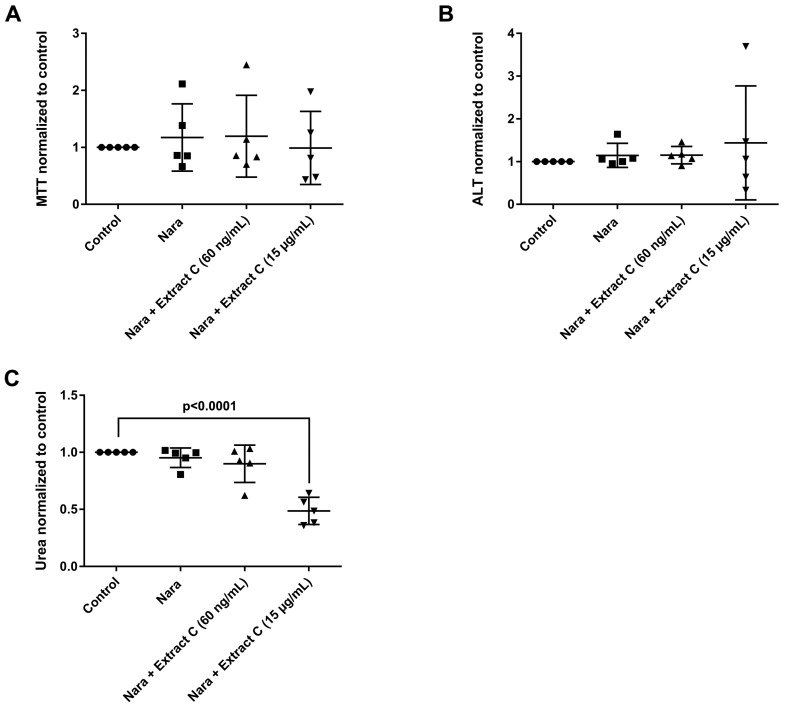
Effects of different *Petasites hybridus* extracts and Naratriptan on clinical liver function tests in cultures of human hepatocytes after daily treatment for 72 h. (**A**) MTT mitochondrial reductase activity. (B) Alanine aminotransferase activity. (**C**) Urea production. The data of individual donors are given normalised to the DMSO vehicle control. The *Petasites hybridus* extracts A, B and C are specified to 37%, <1% and 19.1% petasin, respectively. Extract C is the marketed product Petadolex^®^ and human hepatocyte cultures were treated at therapeutic C_max_ (=60 ng/mL) and >49-fold therapeutic petasin C_max_ (=15 µg/mL) concentrations. Hepatocyte cultures were also treated with Paracetamol at therapeutic C_max_ (=Para; 20 µg/mL) concentrations and in combinations with different *Petasites hybridus* extracts. The data were analysed with the GraphPad Prism software version 6.05.

Liver function tests (LFTs) were evaluated in cultures of primary human hepatocytes (PHH) that were treated with different butterbur extracts (extract A-C) and medications commonly used for migraine attacks. This enabled an assessment of possible drug-herb interactions (Figure 3, Figure 4 and Figure 5). Given that therapeutic Petadolex^®^ concentrations are defined by the petasin content, the data are also expressed relative to therapeutic C_max_ petasin concentrations. Furthermore, hepatocyte CYP monooxygenase activities were evaluated with testosterone as a substrate. No hepatotoxicity was observed at therapeutic C_max_ Petadolex^®^ or Paracetamol concentrations, as determined by the MTT, LDH, ALT, GGT and urea assay (Figure 3A–E). 

The combined Petadolex^®^ and Paracetamol treatment did not cause hepatotoxicity at therapeutic C_max_ concentrations. Similarly, treatment of PHH at therapeutic C_max_ Petadolex^®^ concentrations did not influence CYP monooxygenase activity using testosterone as a substrate (Figure 3F,G). However, at >49-fold therapeutic C_max_ petasin (=active ingredient) concentrations, marked reduction in MTT activity among four donors was noted to indicate burdened mitochondrial metabolism. One donor also presented three-fold increases in ALT. Equally, the combined high dose Petadolex^®^ and Paracetamol treatment caused marked reductions in MTT activity and urea detoxification (Figure 3A,E) as well as ≤9- and 7-fold increases in ALT. Furthermore, LDH was increased up to 14- and 7-fold, respectively, among individual donors exposed to extract A and C (Figure 3B,C). Except for one donor (MTT-assay), extract B did not elicit significant changes in LFTs. Note GGT-activities were too variable to permit meaningful interpretation of the data. Depicted in Figure 3F,G are up to 3- and 7-fold increases in 2α- and 16α-testosterone hydroxylation after treatment of PHH at >49-fold therapeutic C_max_ petasin concentrations. The increased production of these metabolites indicates induced CYP2C8, CYP2C9, CYP2C19 and CYP3A4 monooxygenase activities. A similar 4- and 6-fold increase in CYP-monooxygenase activities was observed at high dose Petadolex^®^ (15 µg/mL), but therapeutic Paracetamol concentrations. Therapeutic C_max_ Paracetamol concentrations alone also elicited induction of 2α-testosterone hydroxylation. 

The combined Petadolex^®^ and Ibuprofen treatment of PHH was well tolerated at therapeutic C_max_ concentrations. The results for cell viability (MTT and LDH assay), ALT, GGT, urea and CYP monooxygenase activities are given in Figure 4. 

At >49-fold C_max_ petasin concentrations, marked reductions in MTT activities were observed. With one donor, the combined treatment with Ibuprofen caused 8- and 7-fold increases in LDH and ALT activities (Figure 4B,C). Individual donors responded with an increased activity in 2α and 16α- testosterone hydroxylation; however, urea detoxification was significantly reduced at 15 µg/mL Petadolex^®^ and therapeutic Ibuprofen concentrations.

Likewise, treatment with Petadolex^®^ and Naratriptan did not impair mitochondrial MTT activity at therapeutic C_max_ concentrations. However, MTT activities were markedly reduced with two donors at >49-fold C_max_ petasin and Naratriptan therapeutic concentrations. One donor presented marked reduction in mitochondrial MTT activity and a small increase in ALT as well as reduced urea measurements at therapeutic Naratriptan concentrations (Figure 5A) to indicate hepatotoxicity (Figure 5). At >49-fold C_max_ petasin and therapeutic Naratriptan concentrations, MTT activity and urea production was reduced (Figure 5A,C); here, one donor presented a nearly four-fold increase in ALT activity (Figure 5B).

## 4. Discussion

Petadolex^®^ has been manufactured for over four decades and was granted market authorization in 1978. It was introduced into the US market in 1998. Unlike many HDS, it was extensively evaluated for safety and efficacy. In clinical studies, the spasmolytic properties and effectiveness in the prevention of migraine attacks was proven [5,6,21]. Therefore, butterbur extracts were recommended for migraine prevention [7]; however, reports of severe HILI cases (8 in Germany, 1 in UK and 1 in Austria) prompted concern regarding its safe use. 

### 4.1. PAs and the Sinusoidal Obstruction Syndrome

Pyrrolizidine alkaloids (PAs) are ingredients commonly found in foods, teas, and medicinal herbs [22,23]. Given the >400 PA species in addition to N-oxides and other derivatives, a comprehensive analysis of PAs is difficult [24]. Butterbur also contains PAs; however, these can be removed to trace amounts during the manufacturing process.

What is the link between PAs and the sinusoidal obstruction syndrome? PAs are metabolically activated by hepatic CYP monooxygenase and sinusoidal endothelium to produce reactive electrophiles capable of forming adducts with cellular macromolecules and DNA [23,25]. Particularly, exposure to higher concentrations of metabolically activated PAs causes SOS and impaired blood flow. SOS is characterized by endothelial cell damage, proliferation and activation of stellate cells, enhanced collagen deposition around central veins, as well as infiltration of activated macrophages within the lumen and the wall of central veins [26]. Endothelial cell injury is considered to be an initiating step in the manifestation of SOS [27]. Clinically, the disease is characterized by hyperbilirubinemia, hepatomegaly, and fluid retention [28].

Several reports on the outcome of human exposure to pyrrolizidine alkaloids describe the risk of SOS. Even childhood liver cirrhosis was reported as a consequence of exposure to pyrrolizidine alkaloids [29]. Notwithstanding SOS might be reversible as reported for an infant exposed to a PA-mixture for 15 months [30]. Case reports associated with PA toxicity include Senecio tea, Gynura root and comfrey [31,32,33]. 

Remarkably, none of the suspected Petadolex^®^ HILI cases were diagnosed with SOS, but presented an inconsistent injury pattern to possibly suggest polypharmacy as a culprit. Similarly, chronic rat toxicity studies did not evidence SOS; however, bile duct proliferation and its dilatation in the absence of significant liver transaminase elevations after dosing for six months [12]. Unlikely clinical cases with a female predilection the preclinical studies with rats suggest males to be more sensitive to excessive dosages. Moreover, the grading of lesions indicated some reversibility of bile duct proliferation after treatment cessation, while explorative studies with minipigs failed to demonstrate morphological changes of the liver at 218-fold MCD. The mechanism of bile duct proliferation following biliary obstruction was the subject of an earlier report [34]. Apparently, increased biliary pressure is an initiating factor. Although hepatocytes and cholangiocytes originate from the same stem cell, the plasticity of hepatocytes and the regulations thereof differ substantially between humans and rodents [35,36]. 

### 4.2. In vitro Hepatotoxicity Testing Provide Mechanistic Clues for Bile Duct Proliferations Observed in Rat Toxicity Studies

At therapeutic C_max_, Petadolex^®^ did not cause hepatocyte toxicity, either given alone or in combination with commonly used migraine medications. However, at supra-therapeutic concentrations, some donors presented changes in LFT and CYP monooxygenase activity after repeated treatment of PHH for 72 h. Given that extract B is low in petasin (<1%) and this extract did not elicit LFT changes, we speculate that excessive petasin concentrations can be harmful to hepatocytes. 

We previously reported regulation of bile acid transporters and solute carriers after treatment of PHH with different butterbur extracts [12]. It is tempting to postulate a relationship between PHH cell culture findings and bile duct proliferations seen in the rat chronic toxicity study. Specifically, ABCB1 was significantly upregulated in PHH and a similar upregulation is observed after bile duct ligation of rats [37]. ABCB1 has broad substrate specificity; its expression is largely dependent on the activity of the Farnesoid X (FXR) bile acid receptor [38]. Conversely, ABCB4 (Mdr2/MDR3) was repressed. This canalicular membrane transporter plays an essential role in the biliary excretion of phospholipids. Furthermore, the sodium/taurocholate co-transporting polypeptide (SLC10A1) was repressed at >49-fold therapeutic Cmax petasin concentrations and functions in bile acid uptake to protect hepatocytes against toxic intracellular bile acid accumulation. Altogether, 18 solute carriers were regulated in PHH and the observed bile duct proliferations seen at 45 and 90-fold MCD might be regarded as adaptive responses to impaired bile transport. 

### 4.3. Pharmacovigilance and the Complexity of Drug Use in Migraine Therapy

Migraine is a complex disease and during attacks patients frequently rely on pain relief medications (NSAIDs, Paracetamol). Equally, the management of migraine pain is multifaceted and next to pain relief medications is a wide range of drugs for prevention therapies, including ß-blockers, Topiramate, Ibuprofen, Amitriptyline and others as summarized by the AHS/AAN [39]. Consequently, it is of no surprise that about half of the suspected Petadolex^®^ HILI cases reported co-medication with drugs known for their liver liabilities. Such co-medication may aggravate risk for HILI [16]. Specifically, an analysis of 54,583 reports of the French Pharmacovigilance database suggested the risk for severe hepatic injury and acute renal failure to be six- to sevenfold higher when two NSAIDs or more were taken [16]. Co-medications in suspected Petadolex^®^ cases involved antibiotics, neuroleptics, antiviral drugs and hormones. Their known liver liabilities may contribute to risk for liver injury [40,41]. In view of the complex pharmacotherapies in some migraine patients, suspected HILI cases may well be the result of co-medication with NSAIDs at least in three out of eight cases, as suggested by the pathologist. Typically, the incidence of hepatobiliary ADRs for NSAIDs is in the range of 2 per 10,000 [31,42]. One study reported an incidence for prescribed drugs of 19.1 cases per 100,000 inhabitants [43]. A Korean study estimated an incidence of HILI at 0.6%; all cases were of the hepatocellular injury type and confined to females [44]. A similar incidence was reported for the Japanese herbal medicine Kampo [45]. To the best of our knowledge no HILI case in association with Petadolex^®^ intake has been reported in the US until today.

### 4.4. Regulatory Issues

The risk for HILI and its possible mechanisms was the subject of an independent review [46]. Likewise, the need for developing better scientific methods to link ingredients of HDS to risk for HILI has been emphasized [47]. Note, in 1988, the manufacturer of Petadolex^®^ changed the extraction solvent from methylene chloride to CO2 to improve the removal of pyrrolizidine alkaloids. In 2002, the German regulatory authority BfArM evaluated this modification in the extraction protocol and concluded that this change would alter the composition of active ingredient. Correspondingly, the product is not the same as the original one that received market authorization and was therefore removed from the market. Likewise, in 2011, the UK Medicines and Healthcare products Regulatory Agency (MHRA) declined an application of the manufacturer for a new traditional license on safety concerns related to the distribution of Petadolex^®^ (alias Migravoid) by retail stores as migraine preventive medication. 

### 4.5. Study Limitations

The following caveats need to be considered. First, under-reporting of adverse drug reactions is a significant problem. Therefore, an estimation of the incidence of hepatobiliary ADRs remains vague. Second, the reported ADRs frequently do not provide comprehensive information on the patient’s medical history, laboratory parameters and a complete listing of concurrent medications. In fact, nearly half of the hepatobiliary ADRs could not be assessed adequately for causality and therefore were judged unclassifiable. A recent review article on the safety of a special butterbur extract also highlighted this problem [48]. Third, it is difficult to perform valid RUCAM assessments only from PSUR data for the reasons described above. Note the causality assessment reported in Table 3 differs from a recently published RUCAM evaluation of severe Petadolex^®^ suspected HILI cases [11]. In the present study, five severe HILI cases were judged as possible, while the published RUCAM assessment considered these as unlikely. This highlights potential pitfalls of RUCAM assessments based on limited PSUR data and reinforces the importance of expert opinion as was summarized in the seminal review of Lewis [49]. 

In conclusion, herbal remedies offer alternative treatments for migraine therapy. The risk for HILI can be minimized by monitoring liver transaminases, especially in patients with polypharmacy and hypersensitivity to the active ingredient or members of the Asteraceae family or any of the excipients. Individuals with a history of pseudoallergic drug reactions, previous liver diseases, alcohol abuse, and familial non-haemolytic hyperbilirubinanaemia (Gilbert’s syndrome) should be carefully monitored, and the concomitant use of St. John’s wort is not recommended.

## Figures and Tables

**Table 1:** Forty-eight spontaneous hepatobiliary ADR case reports in association with Petadolex^®^ intake.

Case No.	Gender (Age)	Diagnosis	Time to Onset of ADRs	Duration of Drug Intake	Daily Dose (mg)	ALT (Max) [×ULN]	AST (Max) [×ULN]	Co-Medications	Notes	WHO/UMC Score	Year of Report
1	Female (34)	Acute hepatitis	~6 weeks	56 days	50	26.20	8.26	Contraceptive, Ibuprofen, Paracetamol, acetylsalicylic acid (Metoclopramide; Fenistil), Hyperforat	-	Possible	1998
2	Female (46)	Acute hepatitis	28 days	34 days	100	6.51	5.29	Sulfasalazine, extract of *peumus boldus*, two homeopathic medications	Comorbidity: Colitis ulcerosa	Possible	1998
3	Female (67)	Minimal transaminase changes	4 days	4 days	75	0.86	0.43	Acetylic salicylic acid, Metoprolol, extract of Silybum marianum	-	Possible	1999
4	Female	Liver enzyme elevation	ni	ni	Ni	ni	ni	ni	Cholecystolithiasis was stated as probable cause for increased liver enzymes by the responsible physician	Unlikely	2000
5	Male	Liver enzyme elevation	ni	ni	Ni	2.83	ni	ni	Elevated transaminases prior to Petadolex^®^ intake	Unclassifiable	2000
6	Female	Liver enzyme elevation	ni	ni	Ni	ni	ni	ni	-	Unclassifiable	2000
7	Male (58)	Acute hepatitis	92 days	115 days	75	32.40	10.97	Zolmitriptan	-	Likely	2001
8	Female (55)	Liver enzyme elevation	~6 months	~6 months	75	ni	ni	High dose pain medications for years	-	Unclassifiable	2001
9	Female (38)	Cholestatic hepatitis	~0.5 months	183 days	75	6.94	8.49	Mestranol and Chlormadinone	-	Unlikely	2001
10	Female (49)	Liver enzyme elevation	~111 days	177 days	100–200	1.74	1.26	Amitriptyline	Previous elevated transaminases were reported for this patients on Amitriptyline monotherapy	Possible	2002
11	Female	Liver enzyme elevation	ni	~5 months	Ni	ni	ni	ni	Elevated transaminases prior to Petadolex^®^ intake	Unlikely	2002
12	Female (45)	Acute hepatitis	~36 days	~36 days	100	28.29	17.14	Ethinylestradiol and Desogestrel	-	Possible	2002
13	Male (14)	Icterus	2 days	11 days	75	ni	ni	ni	-	Possible	2002
14	Female	Liver enzyme elevation	~4 weeks	~15 months	Ni	ni	ni	ni	-	Unclassifiable	2002
15	Female (33)	Minimal transaminase changes	ni	22 days	150	1.18	0.50	ni	Despite discontinuation of Petadolex^®^ treatment transaminases increased; possibly related to chronic medication for polyarthritis	Unlikely	2003
16	Female (47)	Liver enzyme elevation	~4 weeks of the second treatment course	92 + 92 days	Ni	ni	ni	Zolpidem	Normalized laboratory values after discontinuation of treatment	Unclassifiable	2003
17	Female (23)	Acute hepatitis	100 days	109 days	50/75	17.86	5.46	Tetrazepam	-	Unclassifiable	2003
18	Female (42)	Acute hepatitis	124 days	124 days	100	9.69	8.26	ni	-	Likely	2003
19	Female	Hepatitis, elevated GGT	2nd month of second treatment course	6 months + 2 months	Ni	ni	ni	ni	GGT > 3 ×ULN	Unclassifiable	2003
20	Male (7)	Minimal transaminase changes	47 days	55 days	50–100	ni	1.1	ni	-	Likely	2003
21	Female (68)	Liver enzyme elevation, abdominal discomfort	51 days	88 days	150	ni	ni	Tamoxifen, probiotic product (*E. coli*)	-	Possible	2004
22	Female (62)	Minimal transaminase changes	61 days prior to drug intake	~13 months	75–150	1.34	ni	ni	-	Unclassifiable	2004
23	Female (50)	Cholestatic hepatitis	~4 days	ni	50	50.89	ni	Thuja, nettle herbal tincture, garlic horseradish winterformula, ß-caroten, chromium, luffa and euphorbia	-	Unclassifiable	2004
24	Female (58)	Minimal transaminase and GGT changes	384 days	~2 years	225	0.29	0.51	Diclofenac and Misoprostol	GGT: 1.1 ×ULN	Unclassifiable	2004
25	Male	Liver enzyme elevation	ni	ni	75/week	ni	ni	Different homeopathic products, vitamins	Previous episodes of serum biochemistry changes in response to drugs	Possible	2004
26	Female	Mild GGT changes	10 days	ni	150	ni	ni	ni	GGT: 1.6 ×ULN	Unclassifiable	2004
27	Female	GGT enzyme elevation	ni	ni	Ni	ni	ni	ni	-	Unclassifiable	2004
28	Female	Bilirubin elevation, icteric sclera	ni	ni	Ni	ni	ni	ni	Recurrent bilirubin elevations after discontinuation of Petadolex^®^ therapy	Unclassifiable	2004
29	Male	Mild GGT changes	ni	~4 months	Ni	ni	ni	ni	GGT: 1.5 ×ULN	Unclassifiable	2004
30	Female (58)	Hepatopathy, weight loss, mild transaminase changes	248 days	248 days	100–150	2.43	1.28	Zolmitriptan on demand	Ferritin 5 ×ULN; positive autoimmune antibody titer for ANA	Possible	2004
31	Male	Marked GGT enzyme elevation	ni	ni	Ni	ni	ni	ni	GGT: ~15 ×ULN	Unclassifiable	2004
32	Female (40)	Mild transaminase changes	ni	282 days	150	3.17	2.55	ni	Elevated liver enzymes prior to Petadolex^®^ treatment, normalization started under Petadolex^®^ therapy	Unclassifiable	2005
33	ni	Liver enzyme elevation	ni	ni	Ni	ni	ni	ni	-	Unclassifiable	2005
34	Female (65)	Mild GGT changes	ni	9 months	Ni	ni	ni	Zolmitriptan, Trimipramin, homeopathic product	Elevated liver enzymes prior to Petadolex^®^ treatment, GGT: ~2 ×ULN	Unlikely	2005
35	Female (40)	Acute hepatitis	92 days	99 days	150	42.12	59.61	Ibuprofen on demand	-	Possible	2005
36	Female (63)	Moderate liver enzyme elevation	ni	227 days	150	8.00	3.90	Pentosan for bladder pain syndrome	-	Unclassifiable	2005
37	Female	GGT enzyme elevation	Intake of 1 package	ni	Ni	ni	ni	ni	-	Unclassifiable	2005
38	Male	GGT enzyme elevation	ni	ni	Ni	ni	ni	ni	-	Unclassifiable	2006
39	Female (24)	Acute hepatitis, acute liver failure	~3 months	~3.5 months	50	152.86	104.00	ni	Liver transplantation	Possible	2006
40	Male (84)	Minimal transaminase changes	within 4 weeks	~8 months	150	1.34	ni	ni	-	Unclassifiable	2006
41	ni	Hepatitis	ni	ni	Ni	ni	ni	ni	-	Unclassifiable	2006
42	Female (48)	Mild GGT and Bilirubin elevations	37 days	42 days	150	ni	ni	ni	GGT 2.3 ×ULN (23.10.); 2.8xULN (6.11.); 2xULN (30.11.) Bili: 1.26 mg/dL; recovery after discontinuation of treatment	Possible	2007
43	Male (39)	Icteric sclera	ni	7 days	50	ni	ni	ni	Physician suspected parallel infection with Noro-Virus as cause for the icterus	Unlikely	2008
44	Male	Mild transaminase and GGT changes	173 days	~5 months	75	2.30	ni	Sumatriptan, L-thyroxine	Gilbert’s Syndrome, Hepatic steatosis, parallel weight increase, GGT 2.93 ×ULN	Unlikely	2008
45	Female	Liver enzyme and GGT elevations	ni	~5 months	50–75	ni	ni	ni	GGT 3.4 ×ULN	Possible	2008
46	Female	Liver enzyme elevation	ni	ni	Ni	ni	ni	ni		Unclassifiable	2009
47	Male	Liver enzyme elevation	ni	ni	Ni	ni	ni	ni		Unclassifiable	2010
48	Male (53)	Choledocholitiasis, marked bilirubin but mild transaminase changes; Endoscopic Retrograde Cholangiopancreatography and re-ERCP after bleeding	ni	~4 years	Ni	1.04	1.22	Unsupervised pain relief medication for migraine for more than 30 years; occasionally opioids; use of herbal dietary supplements of Traditional Chinese Medicine	Bilirubin > 46-fold elevated, AP > 2-fold, aPTT = 48%, Hyperbilirubinemia resolved slowly with marked *pruritus* and weight loss of > 16 kg in a short period of time, liver biopsy highlights bile flow obstruction but no indication for sinusoidal obstruction syndrome and HILI	Unlikely	2011

ni = no information.

**Table 2:** Twelve non-serious hepatobiliary ADR case reports recorded during clinical trials with 397 patients on Petadolex^®^. Information on 12 hepatobiliary events recorded during clinical studies and suspected to be related to exposure with *Petasites hybridus* extracts.

Case No.	Gender (Age)	Time to Onset of ADRs (days)	Duration of Drug Intake (Days)	Daily Dose (mg)	ALT (Max) [×ULN *]	AST (Max) [×ULN *]	Co-Medications	Notes	WHO/UMC Score	Year of Report
1	Female	3 months	3 months	100	0.80	1.17	Acute migraine medication possible, other unknown	Clinical study [3] Minimal transaminase changes	No clinical relevance	1993
2	Female	3 months	3 months	100	0.69	1.11	Acute migraine medication possible, other unknown	Clinical study [3] Minimal transaminase changes	No clinical relevance	1993
3	Female	3 months	3 months	100	0.63	1.00	Acute migraine medication possible, other unknown	Clinical study [3] Minimal transaminase changes	No clinical relevance	1993
4	Female	4 months	4 months	50	0.66	0.80	L-Thyroxine, Rizatriptan, Cyclandelat, Acetylsalicylic acid + Paracetamol, Sumatriptan	Clinical study [5] Minimal transaminase changes	No clinical relevance	1999
5	Female	4 months	4 months	50	0.59	0.60	L-Thyroxine, Estradiol, Enalapril + Hydrochlorothiazid, Paracetamol, Meatmizol, Ibuprofen, Acetylsalicylic acid, Acetylsalicylic acid + Paracetamol	Clinical study [5] Minimal transaminase changes	No clinical relevance	1999
6	Female	4 months	4 months	50	0.77	0.87	Acetylsalicylic acid, Phenazone/caffeine, Paracetamol, dihydroergotamin, DL-Lysinmono(acetylsalicylat) (Aspisol), Metamizole, Acetylsalicylic acid + Paracetamol	Clinical study [5], Cardiac insufficiency since 1993, Hypertension since 1997, Hypercholesterinaemia, Hyperthyroidism since 1992; GGT approximate 1.3 ×ULN Cardiac arrhythmia since 1998, Bilirubin increased approximately 1.35 ×ULN and recovered in the follow up period	No clinical relevance	1999
7	Male	4 months	4 months	50	0.93	1.26	Albuterol, Salmeterol, Rizatriptan, Lisinopril + Hydrocholorothiazide, Rizatriptan, Acetaminophen	Clinical study [5], chronic obstructive pulmonary disease; Minimal transaminase changes	No clinical relevance	1999
8	Female	4 months	4 months	50	0.63	0.57	Naratriptan, Butabital + Acetaminophen + caffeine	Clinical study [5], GGT (1.52 ×ULN)	Possible	1999
9	Male	4 months	4 months	75	1.09	0.83	Naratriptan, Acetylsalicylic acid, Metoclopramide	Clinical study [5] Minimal transaminase changes	No clinical relevance	1999
10	Male	4 months	4 months	75	1.23	0.95	Sumatriptan, Rizatriptan	Clinical study [5], GGT at screening: 5 ×ULN; final visit 5.24 ×ULN)	Unlikely	1999
11	Female	4 months	4 months	75	0.48	0.26	Nabumetone, Loratidine, Albuterol, Acetaminophen, Acetaminophen + Acetylsalicylic acid + caffeine	Clinical study [5] Minimal transaminase changes	No clinical relevance	1999
12	Female	4 months	4 months	75	1.05	0.71	Norethindrone, Ethinyl Estradiol, Celecoxib, Acetaminophen + Acetylsalicylic acid + caffeine, Ibuprofen	Clinical study [5] Minimal transaminase changes	No clinical relevance	1999

* Reference values of the clinical study centres: Diener et al. [3] and Grossmann et al. [4]: AST ≤ 18 U/L, ALT ≤ 17 U/L; Lipton et al. [5]: AST ≤ 42 U/L, ALT ≤ 40 U/L.

**Table 3:** A summary of 10 severe HILI cases in association with Petadolex^®^ intake.

Case No.	Diagnose	Duration of Treatment	Reported Symptoms	Course and Outcome	Laboratory Parameters	Additional Findings	WHO/UMC Score
1	Necrotizing cholestatic hepatitis	~2 months	Feeling sick, pruritus, scleral jaundice, eczema, elevated serum transaminases	After cessation of treatment laboratory parameters returned to normal	- Lymphocyte-proliferation assay pos. with an index value of 3 for Petadolex^®^ - HIV, EBV, hepatitis serology neg. - Listeria monocytogenes & *Toxoplasma gondii* neg. - M. Wilson & haemochromatosis negative	Amenorrhoea	Possible
7	Cholestatic hepatitis	115 days	Diffuse abdominal discomfort and scleral jaundice	Improved condition after cessation of treatment	EBV cannot be excluded as possible cause, since IgM antibodies were positive, other serology negative	-	Likely
9	Cholestatic hepatitis	~6 months	Discomfort, jaundice, elevated cholestasis parameters and bilirubin, progressing symptoms	Liver transplantation	Autoantibodies negative, M. Wilson negative, mechanic cholestasis excluded	Hepatitis onset 6 weeks after Petadolex^®^ treatment discontinuation	Unlikely
12	Hepatitis of unknown origin	~6 weeks	Elevated transaminases, abdominal discomfort, jaundice, cholestasis, brown colored urine and discoloration of stool, sonographic signs of cholecystitis, cholecystolithiasis and polyps	No information	ANA titer positive, Hep A and B negative, Antibodies against Hep C; coeruloplasmin and serum copper within normal range to dismiss M. Wilson as possible cause; no reported alcohol abuse	-	Possible
17	Hepatitis	~3.5 months	Hospital admission with nausea, emesis and abdominal discomfort, liver enzyme elevations, weight loss of 15 kg in 10 months with onset before treatment had started	Treatment was stopped and laboratory values returned to normal. Six months later, the patient presented with thoracic pain, physical and laboratory parameters revealed no pathophysiological findings. Psychological counselling was suggested	Anti-HAV-IgM neg. Hep B neg. Hep C neg.	Medical history of recurrent thoracic pain episodes; no indications for a cardiac cause; paresis of the N. facialis, Factor-V-Leyden mutation, Splenomegaly	Unclassifiable
23	Cholestatic hepatitis	No information	Abdominal discomfort, nausea, jaundice, pruritus, dark colored urine, liver enzyme elevations	No information	Hep A neg. Hep B neg. Anti- HCV neg.	Medical history with polypectomy, blood transfusion, *rhinitis allergica*	Unclassifiable
30	Hepatopathy	248 days	Elevated liver enzymes, weight loss, liver biopsy was taken	Normalization of transaminases after cessation of treatment	Elevated ANA titer: 1:320; ferritin (5 ×ULN), haemochromatosis gene (HFE) testing negative	-	Possible
35	Acute hepatitis	99 days	Colored urine, jaundice of skin- and sclera, abdominal discomfort, inappetence		Status after Hep A infection, EBV IgG pos, CMV pos. autoantibodies negative, exclusion of M. Wilson, haemochromatosis & AT1 deficiency	Medical history of pyelonephritis, appendectomy, *Sectio caesarea*, no alcohol abuse	Possible
39	Acute liver failure	4 month	Fatigue, inappetence, jaundice of skin and sclera, after hospitalization admission liver failure, encephalopathia grade III	After 8 days of hospitalization liver transplantation	ANA titer slightly elevated: 1:80, anti-neutrophil cytoplasmic antibodies (x-ANCA) 1:160 elevated, other autoantibody titers negative, exclusion of M.Wilson; marked elevation of ferritin and serum iron levels	Medical history: acute hepatitis with jaundice in 1997 (acute lymphocytic hepatitis), 2 months after tonsillectomy (DD: Fluran -induced hepatitis)	Possible
48	Cholestatic hepatitis, choledocholitiasis	~4 years	Marked pruritus and weight loss of > 16 kg in a short period of time, liver biopsy highlights bile flow obstruction but no indication for sinusoidal obstruction syndrome and HILI	Endoscopic Retrograde Cholangio-pancreatography and re-ERCP after bleeding; hyperbilirubinemia resolved slowly	Bilirubin > 46-fold elevated, AP > 2-fold, aPTT = 48%	Unsupervised pain relief medication for migraine for > 30 years; occasionally opioids; use of herbal dietary supplements of Traditional Chinese Medicine	Unlikely

## Supplementary Materials

The following are available online at https://www.mdpi.com/2077-0383/8/5/652/s1, Figure S1: Serum biochemistry changes (ALT, AST, GGT) in regards to dose and time to onset of HILI, Table S1: Information on individual human donors, which donated liver tissue for the generation of human hepatocyte cultures, Table S2: Clinical biochemistry data and major histopathology findings after repeated oral dosing of rats with a *Petasites hybridus* extract for six months.
