# Supplementary material for: Hepatobiliary Events in Migraine Therapy with Herbs—The Case of Petadolex, A Petasites Hybridus Extract
Source: J Clin Med. 2019 May 10;8(5):652. doi: 10.3390/jcm8050652 (PMC6572430; doi:10.3390/jcm8050652)
Supplement: Supplementary file 1 [file jcm-08-00652-s001.pdf]

SUPPLEMENTARY DATA

Hepatobiliary events in migraine therapy with herbs – The case of Petadolex, a Petasites hybridus extract

Nora Anderson and Jürgen Borlak

Hannover Medical School, Centre for Pharmacology and Toxicology, Carl-Neuberg-Str. 1, 30625 Hannover, Germany

E-mail: Borlak.Juergen@mh-hannover.de

Supplementary Figure S1. Serum biochemistry changes (ALT, AST, GGT) in regards to dose and time to onset of HILI.

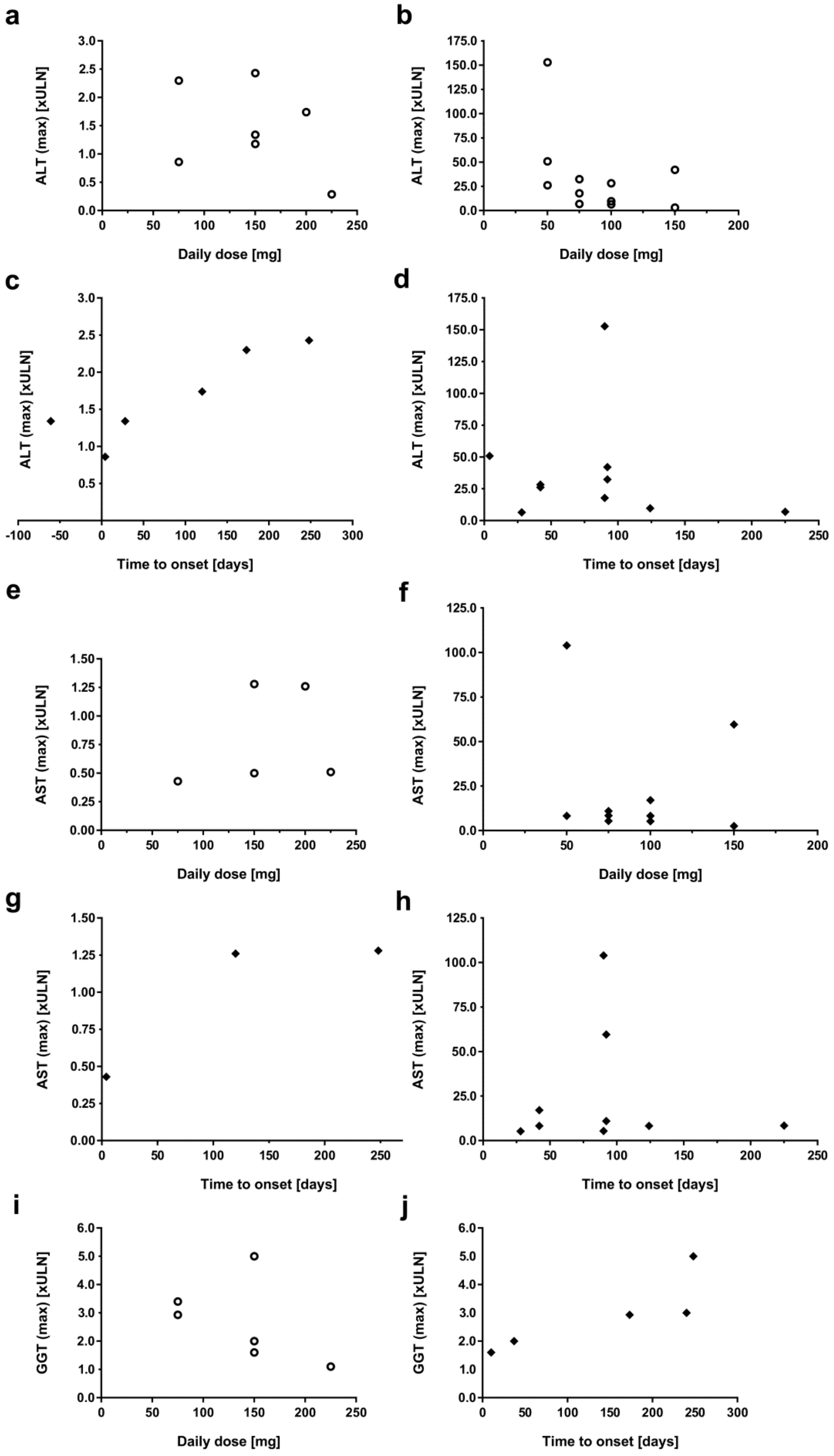

Panel A: ALT in relation to dose ≤ 3x ULN

Panel B: ALT in relation to dose > 3x ULN

Panel C: ALT in relation to time to onset ≤ 3x ULN

Panel D: ALT in relation to time to onset > 3x ULN

Panel E: AST in relation to dose ≤ 3x ULN

Panel F: AST in relation to dose > 3x ULN

Panel G: AST in relation to time to onset ≤ 3x ULN

Panel H: AST in relation to time to onset > 3x ULN

Panel I: GGT in relation to dose

Panel J: GGT in relation to time to onset

Supplementary Table S1: Information on individual human donors, which donated liver tissue for the generation of human hepatocyte cultures.

Donor ID	Sex	Age	Diagnosis
**1**	Male	67	Klatskin Tumor
**2**	Male	74	Liver metastasis of colorectal carcinoma
**3**	Male	69	Liver metastasis of colorectal carcinoma
**4**	Male	57	Liver metastasis of colorectal carcinoma
**5**	Female	77	Liver metastasis of ovarian tumor
**6**	Male	57	Liver metastasis of colorectal carcinoma
**7**	Male	36	Liver metastasis of colorectal carcinoma
**8**	Female	41	Liver metastasis of ovarian tumor
**9**	Male	50	Liver metastasis of colorectal carcinoma

Supplementary Table S2: Clinical biochemistry data and major histopathology findings after repeated oral dosing of rats with a Petasites hybridus extract for 6 months.

Study group	Vehicle control		45mg/kg		135 mg/kg		270 mg/kg	
gender	M	F	M	F	M	F	M	F
**Serum biochemistry**								
AST*	30.05 (±1.52)	32.60 (±2.47)	32.30 (±2.80)	29.50 (±4.06)	29.40 (±1.06)	27.10 (±1.15)	26.48 (±5.46)	25.88 (±3.15)
Bilirubin*	1.81 (±0.59)	2.03 (±0.43)	2.04 (±0.86)	2.13 (±0.74)	2.48 (±0.97)	2.15 (±0.92)	3.35 (±1.74)	2.13 (±0.76)
ALAT*	18.23 (±1.65)	15.58 (±2.89)	20.09 (±0.95)	19.17 (±5.73)	20.40 (±2.10)	16.60 (±2.38)	15.43 (±.1.71)	13.13 (±1.23)
GGT *	0.68 (±0.53)	0.68 (±0.61)	1.03 (±0.78)	0.77 (±0.15)	1.13 (±0.25)	0.83 (±0.55)	2.10 (±1.66)	2.85 (±2.15)
AP *	162.78 ±82.07	111.23 ±53.88	180.10 ±87.04	87.87 ±47.33	179.43 ±84.68	93.20 ±43.68	156.70 ±83.40	89.35 ±41.81
**Histological findings**								
Focal lymphocytosis ** (number of animals)	0	2	4	4	5	4	1	3
Bile duct hyperplasia** (number of animals)	0	0	0	0	10	5	17	13
Bile duct dilatation** (number of animals)	0	0	0	0	0	0	16	3
Minimal lipidosis** (number of animals)	7	4	5	4	4	3	9	6

* 4 animals per dose and gender; data are units per liter
** 20 animals per dose and gender; data are individual animals with a histological finding
